# Photosynthetic assimilation of ^14^C into amino acids in potato (*Solanumtuberosum*) and asparagine in the tubers

**DOI:** 10.1007/s00425-013-1967-0

**Published:** 2013-10-15

**Authors:** Nira Muttucumaru, Alfred J. Keys, Martin A. J. Parry, Stephen J. Powers, Nigel G. Halford

**Affiliations:** 1 Plant Biology and Crop Science Department, Rothamsted Research, Harpenden, Hertfordshire AL5 2JQ UK; 2 Computational and Systems Biology Department, Rothamsted Research, Harpenden, Hertfordshire AL5 2JQ UK

**Keywords:** Acrylamide, Amino acid transport, Crop quality, Food safety, Nitrogen transport, Free amino acids, Maillard reaction, *Solanum*, Thin-layer chromatography

## Abstract

Asparagine is the predominant free amino acid in potato tubers and the present study aimed to establish whether it is imported from the leaves or synthesised in situ. Free amino acid concentrations are important quality determinants for potato tubers because they react with reducing sugars at high temperatures in the Maillard reaction. This reaction produces melanoidin pigments and a host of aroma and flavour volatiles, but if free asparagine participates in the final stages, it results in the production of acrylamide, an undesirable contaminant. ^14^CO_2_ was supplied to a leaf or leaves of potato plants (cv. Saturna) in the light and radioactivity incorporated into amino acids was determined in the leaves, stems, stolons and tubers. Radioactivity was found in free amino acids, including asparagine, in all tissues, but the amount incorporated in asparagine transported to the tubers and stolons was much less than that in glutamate, glutamine, serine and alanine. The study showed that free asparagine does not play an important role in the transport of nitrogen from leaf to tuber in potato, and that the high concentrations of free asparagine that accumulate in potato tubers arise from synthesis in situ. This indicates that genetic interventions to reduce free asparagine concentration in potato tubers will have to target asparagine metabolism in the tuber.

## Introduction

Interest in the synthesis, transport and accumulation of free amino acids, particularly free asparagine, has been stimulated in recent years by the discovery of acrylamide in common, plant-derived foods (Tareke et al. 2002). Acrylamide (CH_2_=CH–CONH_2_) is classified as probably carcinogenic to humans, based on its carcinogenic action in rodents; it also has neurological and reproductive effects (Friedman 2003). The FAO/WHO Expert Committee on Food Additives has recommended that dietary exposure to acrylamide should be reduced (Joint FAO/WHO Expert Committee of Food Additives 2011) and the European Commission issued ‘indicative’ levels of acrylamide in food in early 2011 (European Food Safety Authority 2011a). Indicative values are not safety thresholds, but are intended to indicate the need for an investigation into why the level has been exceeded. The food industry has devised many strategies for reducing acrylamide formation by modifying food processing (compiled in a ‘Toolbox’ produced by Food Drink Europe: http://www.fooddrinkeurope.eu/uploads/publications_documents/Toolboxfinal260911.pdf), and in Europe this has resulted in a significant downward trend for mean levels of acrylamide in potato crisps from 763 ppb in 2002 to 358 ppb in 2011, a decrease of 53 % (Powers et al. 2013).

Acrylamide is formed in the Maillard reaction, a complex series of non-enzymic reactions involving reducing sugars and amino groups. The Maillard reaction was named after French chemist, Louis Camille Maillard, who first described it in 1912, although many of the steps were elucidated later by an American chemist, John Hodge. It is also responsible for the production of melanoidin pigments and a plethora of aroma and flavour volatiles, including heterocyclic compounds such as pyrazines, pyrroles, furans, oxazoles, thiazoles and thiophenes (Mottram 2007; Halford et al. 2011). Amino acids participate in the first part of the reaction, which begins with the condensation of the carbonyl (C=O) group of the reducing sugar with an amino group and gives rise to sugar dehydration and fragmentation products containing one or more carbonyl groups. They also react with these carbonyl compounds and one of these reactions is Strecker degradation, whereby an amino acid is deaminated and decarboxylated to give an aldehyde. It is a Strecker-type reaction involving asparagine that gives rise to acrylamide (Mottram et al. 2002; Stadler et al. 2002; Zyzak et al. 2003).

Potato (*Solanum tuberosum*) products are major contributors to dietary exposure to acrylamide in Europe (European Food Safety Authority 2011b, 2012) and there is now strong pressure from the food industry for the development of varieties with reduced acrylamide-forming potential. The relationship between asparagine, sugars and acrylamide formation in potato products is complicated. Asparagine typically accounts for approximately one-third of the total free amino acid pool (Eppendorfer and Bille 1996; Oruna-Concha et al. 2001; Amrein et al. 2003; Elmore et al. 2007; Carillo et al. 2012; Halford et al. 2012a; Muttucumaru et al. 2013) and because asparagine is present at such a high concentration, sugar concentrations might be expected to be the limiting factor for acrylamide formation. Some studies have shown this to be the case (Amrein et al. 2003; Becalski et al. 2004; de Wilde et al. 2006), but others have found asparagine concentration per se or asparagine concentration expressed as a proportion of the total free amino acid pool to be important as well (Elmore et al. 2007, 2010; Shepherd et al. 2010; Halford et al. 2012a). This is discussed in detail elsewhere (Muttucumaru et al. 2008; Halford et al. 2012b), but it is clear that differences in precursor concentration do not affect the reaction in the way that they would if the reaction were a simple one. Nevertheless, concentrations of sugars, free asparagine and other amino acids can have a profound effect on the acrylamide-forming potential of potatoes and current advice is that all of these parameters should be considered in variety selection and as targets for crop improvement.

The metabolic pathways for the synthesis of all of the protein amino acids are present in leaves (Foyer et al. 2003), where reduced nitrogen is readily available from the reduction of nitrate through nitrite to ammonia. Ammonia is assimilated through the glutamine synthetase-glutamate synthase (glutamine 2-oxoglutarate amino transferase) or GS-GOGAT cycle. First, ammonia and glutamate react to form glutamine, catalysed by glutamine synthetase (GS), then the amide group of glutamine is transferred to 2-oxoglutarate to produce two glutamate molecules, catalysed by glutamate synthase (GOGAT) [reviewed in detail by Lea and Azevedo (2007) and Forde and Lea (2007)]. The α-amino group of glutamate may be transferred to oxaloacetate by aspartate aminotransferase to make aspartate and the transfer of the amide group of glutamine to aspartate by asparagine synthetase generates glutamate and asparagine.

In leaves, photosynthetic assimilation of ^14^C results in labelling of soluble amino acids and protein. In C_3_ plants, glycine and serine become labelled rapidly and are saturated with ^14^C in about 15 min in ambient air, because of their rapid turnover in the photorespiratory nitrogen cycle (Ongun and Stocking 1965; Mahon et al. 1974; Kumarasinghe et al. 1977), but, with time, all of the soluble amino acids become radioactive. In contrast, in the absence of significant pools of nitrate, it has been proposed that reduced nitrogen arrives in the tubers mainly in the form of amino acids translocated from the leaves (Millard et al. 1989). Asparagine is used as a major nitrogen storage and transport molecule in many plant species because it is relatively inert and has a relatively high N:C ratio (2:4) compared with other amino acids (Lea et al. 2007). Asparagine synthetase has, therefore, been proposed to be a key enzyme in the control of N transport and its overexpression in transgenic Arabidopsis causes an increase in asparagine concentration in the phloem (Lam et al. 2003).

Asparagine accumulates to very high levels in the grain of wheat plants that are starved of sulphur (Muttucumaru et al. 2006; Granvogl et al. 2007; Curtis et al. 2009), although the same response does not occur in rye, at least under field conditions (Postles et al. 2013). Wheat may use free asparagine as an alternative nitrogen store when a lack of sulphur reduces storage protein synthesis, and asparagine synthetase gene expression in the leaves increases approximately eightfold in response to sulphur deficiency (Byrne et al. 2012). This response can be disrupted by the over-expression of a general control non-derepressible-2 (GCN2)-type protein kinase (Byrne et al. 2012), which is involved in the regulation of protein and amino acid biosynthesis. Some potato varieties also accumulate asparagine in response to very severe sulphur deficiency in pot experiments but others accumulate glutamine instead (Elmore et al. 2007) and the effect has not been demonstrated in the field (Muttucumaru et al. 2013).

The development of low free asparagine potato varieties will require a good understanding of asparagine synthesis, metabolism and transport, but, perhaps surprisingly, data on this aspect of potato physiology are sparse. The major site of synthesis of asparagine in potato remains to be identified, and the relative contribution of asparagine import from the leaves compared with synthesis in situ has not been determined for tuber asparagine accumulation. In the present study, therefore, we have used ^14^C labelling to identify the free amino acids being synthesised in potato leaves and transported via the stem and stolon to developing tubers. The experiment was designed to test whether asparagine in the tuber is derived from translocated asparagine made in the leaf.

## Materials and methods

### Growing of potato plants

Initially, a potato (*S. tuberosum*) cv. Saturna plant (Sutton Bridge Crop Storage Research, UK) was grown in a glasshouse in a pot containing perlite to enable easy sampling of the tubers. Day temperature was maintained at 18 °C and night temperature at 16 °C; supplementary lighting was used to provide the plants with a 16-h day. From the time of shoot emergence, the plants were watered with nutrient solution containing 0.52 g L^−1^ KCl, 0.14 g L^−1^ KH_2_PO_4_, 0.47 g L^−1^ CaNO_3_·4H_2_O, 0.41 g L^−1^ Mg(NO_3_)_2_·6H_2_O, 0.006 g L^−1^ NaCl, 0.44 g L^−1^ CaCl_2_, 0.28 g L^−1^ MgSO_4_·7H_2_O, 0.02 g L^−1^ FeNa-EDTA and trace amounts of copper, zinc, manganese, ammonium molybdate and boric acid.

In a subsequent experiment, three tubers were planted per 11 L pot in compost, with buds removed so that each tuber produced only a single shoot (Chen and Setter 2012). When the shoots were approximately 15 cm tall, the mother tubers were removed and individual plants were transferred to a new 11 L pot. The plants were kept in a controlled environment cabinet with short (10 h) days to encourage tuber formation. Two plants were transferred back to the glasshouse for 2 days before exposure of the leaves to ^14^CO_2_. In the first experiment, this was performed 7 weeks after planting, while in the second experiment, it was performed 53 days after planting.

### Supplying ^14^CO_2_ to potato plants

For the first experiment, NaH^14^CO_3_, 1,850 kBq (50 μCi) (Amersham Biosciences, Amersham, UK) was added to a vial fitted with a septum and containing 90 μL 0.1 M NaH^12^CO_3_ to give a final activity of 185 kBq/μmol (5 μCi μmol^−1^). Sulphuric acid (0.1 mL, 2 M) was injected into the vial to generate ^14^CO_2_, which was flushed through a hypodermic needle to a tube leading to a double-walled (2 × 500 gauge) polyethylene storage bag by injection through another needle of 400 mL of CO_2_-free air. The tube leading to the bag containing the ^14^CO_2_ in 400 mL of air was clamped and the bag transported to the glasshouse and supported on a stand close to the potato plant. A polyethylene bag was clamped around a leaf, approximately 60 cm from the base of the stem, so that the plastic was sealed round the petiole by pressure from a sponge plastic strip pressed against the outside by wooden batons. This bag was quickly evacuated and filled, during 30 min, with the 400 mL of gas containing the ^14^CO_2_.

The initial concentration of CO_2_ was intended to be above ambient, at approximately 560 μL L^−1^, and the mean photosynthetic photon flux density at the leaf surface was estimated as 250 μmol m^−2^ s^−1^ using a Quantum Sensor (Lambda Laboratory Instruments, Brno, Czech Republic). The bag containing the ^14^CO_2_ was left around the leaf for a further 3 h, after which the gas in the bag was transferred back into the storage bag and later drawn into an evacuated flask containing 0.5 mL of 0.5 M NaOH. Internodal stem sections were harvested from the base of the stem upwards to the assimilating leaf. Two leaves above and below the assimilating leaf were also harvested, as well as tubers (starting with the tuber immediately below the assimilating leaf, then proceeding clockwise around the base of the stem), stolons and root tissue. The harvested tissues were placed in 50 mL falcon tubes, frozen in liquid nitrogen and stored at −80 °C before being freeze-dried and powdered.

For the second experiment, a solution containing 30 MBq and 81 μmol of NaH^14^CO_3_ (370 kBq μmol^−1^) was injected into 0.4 mL of 3.5 M lactic acid through which was bubbled CO_2_-free air at 200 mL min^−1^. The generated gas (approximately 2 L) was collected in a double-walled polythene storage bag for 10 min. Two single-stem plants were selected and the tops enclosed in bags using double-sided tape to secure the mouth either side of the stem and a mastic material to create a seal. Air was partially removed from the bags around the two plants and replaced with approximately equal volumes of the air from the storage bag containing the ^14^CO_2_. After 1 h, as much as possible of the content of each bag around the plants was transferred back to the storage bag. The bags were then carefully removed and the plants left in the glasshouse for a further 2.5 h before being removed from the pots. Leaves and stems that had been enclosed in the bag, stem sections, stolons and tubers were flash-frozen in liquid nitrogen and freeze-dried.

### Measurement of ^14^C in crude plant extracts and freeze-dried powders

Samples of the freeze-dried powdered tissues were suspended in 80 % ethanol (50 mg per mL), and the suspensions heated at 70 °C for 5 min and centrifuged at 14,000*g* for 5 min. Supernatant (5 μL) was mixed with 0.4 mL water and 3.0 mL scintillation fluid in 6 mL vials and the ^14^C measured in a liquid scintillation analyser (Perkin Elmer Tricarb 2,100 TR, Beaconsfield, UK). Samples that contained detectable amounts of ^14^C were taken forward for analysis by thin-layer chromatography (TLC). In the first experiment, these were from the stolons and tuber directly below the assimilating leaf, the stem below the assimilating leaf and the assimilating leaf itself. In the second experiment, in which multiple leaves were involved in assimilation, ^14^C in the freeze-dried powders was measured by suspending small samples in 4 mL of Ultima Gold scintillation fluid (Perkin Elmer) with the aid of 160 mg of Cab-O-Sil (silica) (Cabot Corporation, Billerica, MA, USA). Here, ^14^C was detectable in all of the tubers and in roots, as well as in stems and assimilating leaves.

### Purification of extracts and elution of amino acids

Amino acids were purified from the crude 80 % ethanol extracts especially to remove sucrose and salts which would interfere with the TLC. Samples from the extracts were dried down under vacuum and the residue re-suspended in 0.5 mL of 0.1 M HCl, mixed well to dissolve the amino acids and loaded onto a 0.5 mL Dowex 50 H^+^ column (Sigma-Aldrich, Poole, UK). The column was washed with 2 mL water and the amino acids eluted with 2 M NH_4_OH. The sample and water were added to the column in aliquots of 0.5 mL, each time collecting 0.5 mL eluate. The fractions containing ^14^C amino acids were combined, dried down and the residues dissolved in a total volume of 100 or 20 μL of 80 % ethanol. Radioactivity in the liquid passing through the column in the water wash, and before the amino acids were eluted, was measured. This would include radioactivity in sugars, sugar phosphates and carboxylic acids.

### Thin-layer chromatography

Chromatography was carried out on 20 cm × 20 cm cellulose layers on glass plates (Merck) in glass TLC chromatanks (Shandon Scientific Ltd., Runcorn, UK). Loadings for the first experiment were 20 μL for the assimilating leaf sample, 40 μL for the stem, 45 μL for the stolon and 50 μL for the tuber sample, taken from a total sample volume of 100 μL. For the second experiment, loadings were 4 μL for the shoot and 10 μL for the tuber sample, from a total sample volume of 20 μL. Chromatograms were developed in the first dimension with *n*-butanol/acetone/diethylamine/water (20:20:3:10, by vol.) (Hodisan et al. 1998) and twice in the second dimension with *n*-butanol/acetone/acetic acid/water (35:35:7:23, by vol.). In between developments, the plates were dried in a stream of air. Finally, the plates were dried again and amino acids detected by spraying with 0.05 % (w/v) ninhydrin solution in 80 % (v/v) ethanol and incubated at room temperature in the dark in a moist atmosphere overnight. The relative position of the separated amino acids was established by a series of chromatograms each with a different combination of known standards (30–300 nmol). The essential feature was that the system should solve the problem of separating asparagine from glutamine, glutamic acid and aspartic acid.

Duplicate chromatograms were prepared for each of the extracts of potato tuber, stem sections, stolon and leaf. Chromatograms were either exposed to X-ray film or used to measure the incorporation of ^14^C in each amino acid by scintillation counting. For scintillation counting, cellulose powder containing individual amino acids shown by ninhydrin staining was scraped from the glass plate, suspended in 3 mL Ultima Gold scintillation fluid (Perkin Elmer), and analysed using a Perkin Elmer TriCarb 2100 TR liquid scintillation analyser.

### Statistical analysis

The method of residual maximum likelihood (REML) was used to fit a linear mixed model to the data from the second experiment, which had replication (two plants), considering the design effects of plant, plant part and measurement within each plant part as random effects, and the main effects and interaction between tissue and amino acids as fixed effects in the model. Appropriate means in statistically significant (*P* < 0.05, *F* test) fixed terms in the model were compared using least significant difference (LSD) values at the 5 % level of significance. A natural log (to base *e*) transformation was applied to account for heterogeneity of variance across the tissue by amino acid combinations. The GenStat (2013, 16th edition, © VSN international, Hemel Hempstead, UK) statistical system was used for this analysis.

## Results

### Assimilation of ^14^C

^14^CO_2_ was supplied to a potato (cv. Saturna) plant 10 weeks after planting, before the leaves had begun to senesce and while tubers were still developing. Different tissues were sampled and analysed by scintillation counting (Table 1) to give guidance on which tissues should be sampled for amino acid analysis. In this first experiment, where ^14^C was supplied to a single leaf, the quantity of label reaching the tubers was found to be rather small. Consequently, in a subsequent experiment with two separate plants, more ^14^CO_2_ with higher specific radioactivity was supplied to plastic bags enclosing all of the leaves on each plant. This resulted in increased ^14^C in the tubers (Table 1). In this experiment, the assimilation and analysis took place 53 days after planting.

**Table 1:** Distribution of ^14^C between different organs of potato (*Solanum tuberosum*) cv. Saturna plants after assimilation of ^14^CO_2_

	Experiment 1	Experiment 2	
		Plant 1	Plant 2
	^14^C (kBq)	^14^C (MBq)	
Total ^14^C assimilated	466.76	11.426	10.577
Organ			
Assimilating leaf/leaves	302.50	10.682	9.806
Tuber 1	65.33	0.054	0.156
Tuber 2	0.18	0.054	0.015
Tuber 3	0.11	0.026	0.043
Tuber 4	5.26	0.052	0.018
Tuber 5	0.71	0.051	0.192
Tuber 6	0.11	0.110	0.054
Tuber 7	6.75	0.031	0.015
Tuber 8	11.14	0.008	
Tuber 9	1.30		
Tuber 10	0.00		
Stolons	7.29	0.011	0.009
Roots	0.45	0.109	0.064
Stem	63.40	0.116	0.108
2 leaves above assimilating leaf	1.65		
2 leaves below assimilating leaf	0.59		

Results are shown from an experiment where a single leaf on one plant was exposed to ^14^CO_2_ and from an experiment with two separate plants where all of the leaves were enclosed in a bag with ^14^CO_2_. Tubers were numbered clockwise around the base of the stem. In the first experiment, the radioactivity appeared predominantly in the tuber immediately below the exposed leaf (Tuber 1) whereas the tubers were more evenly labelled in the subsequent experiment with two plants. Note the different units for the two experiments

The potential effect on photosynthesis of enclosing the leaf or leaves in the plastic bags was considered in the experimental design. The initial concentration of CO_2_ was above 500 μL L^−1^ and during the main period of assimilation it would have been above ambient. Enough time had to be allowed for the transfer of sufficient radioactivity to the tubers to ensure that amino acids in the plant tissues could be detected without overloading the chromatographic system. If CO_2_ approached the compensation concentration, an increased flux of carbon into glycine and serine would have been expected, but this would have been late in the period during which the leaf was enclosed in the bag. Light intensity in the growth room was kept low to prevent the temperature inside the bag from rising.

### Separation of free amino acids by thin-layer chromatography

**Fig. 1:**
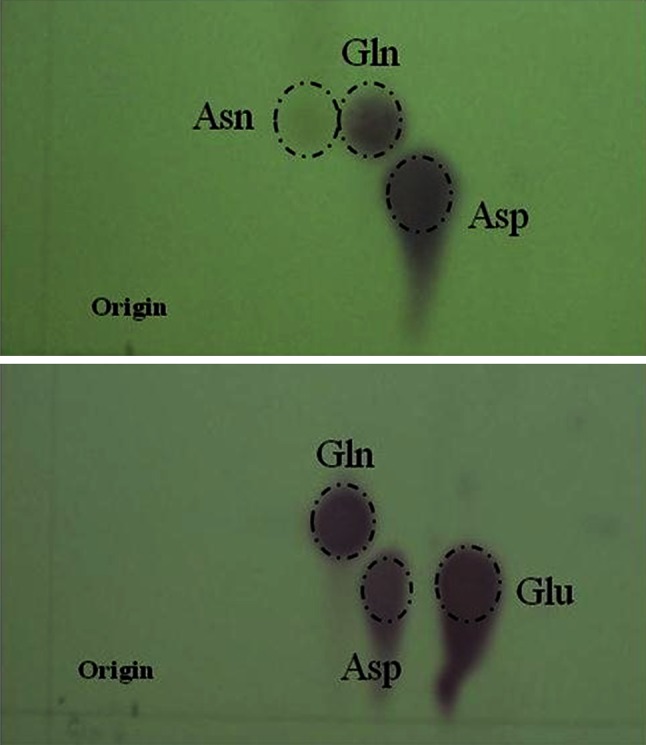
Separation of asparagine, glutamine and aspartate (*top panel*) and of glutamine, aspartate and glutamate (*bottom panel*) by two-dimensional thin-layer chromatography (TLC). Loading for asparagine, glutamine and glutamate was 60 nmol, while for aspartate it was 120 nmol. Chromatography was carried out in solvents 1 [*n*-butanol, acetone, diethylamine and water in ratios (v/v) of 20:20:3:10 (Hodisan et al. 1998)] and 2 (*n*-butanol, acetone, acetic acid and water in ratios (v/v) of 35:35:7:23) (twice in the same direction). The amino acids were stained by spraying the plate with ninhydrin solution (0.05 % in 80 % ethanol)

**Fig. 2:**
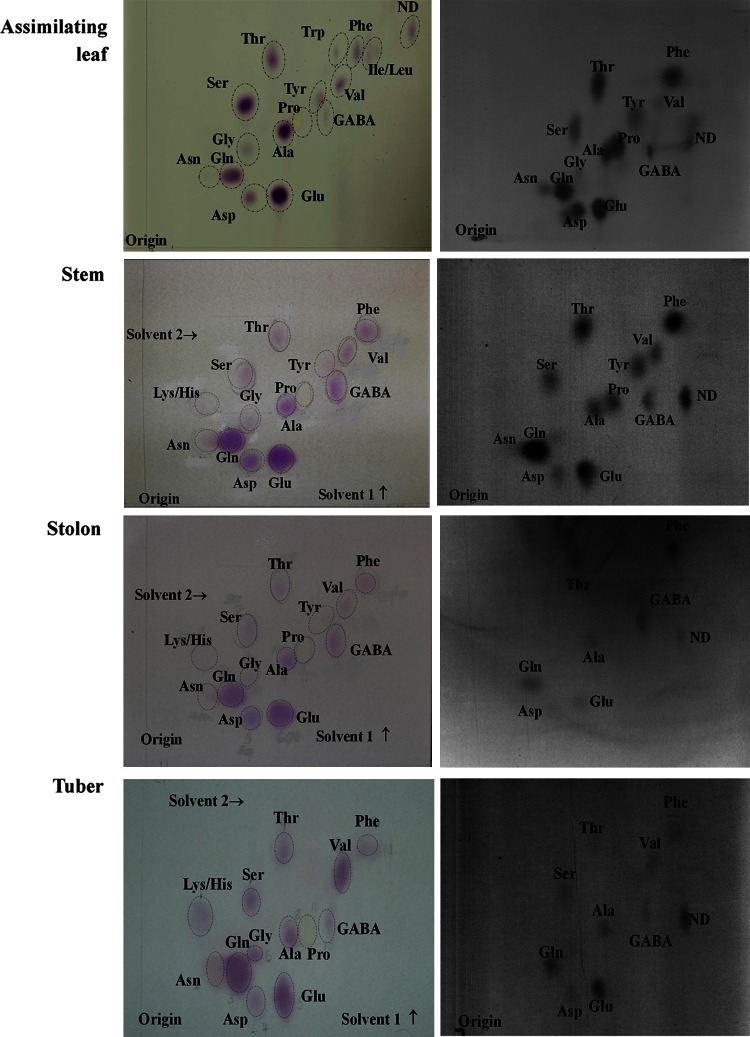
Thin-layer chromatography of amino acids extracted from different tissues of a potato (*Solanum tuberosum*) cv. Saturna plant after assimilation of ^14^CO_2_. Chromatograms stained with ninhydrin are shown on the *left* and autoradiographs on the *right*

**Fig. 3:**
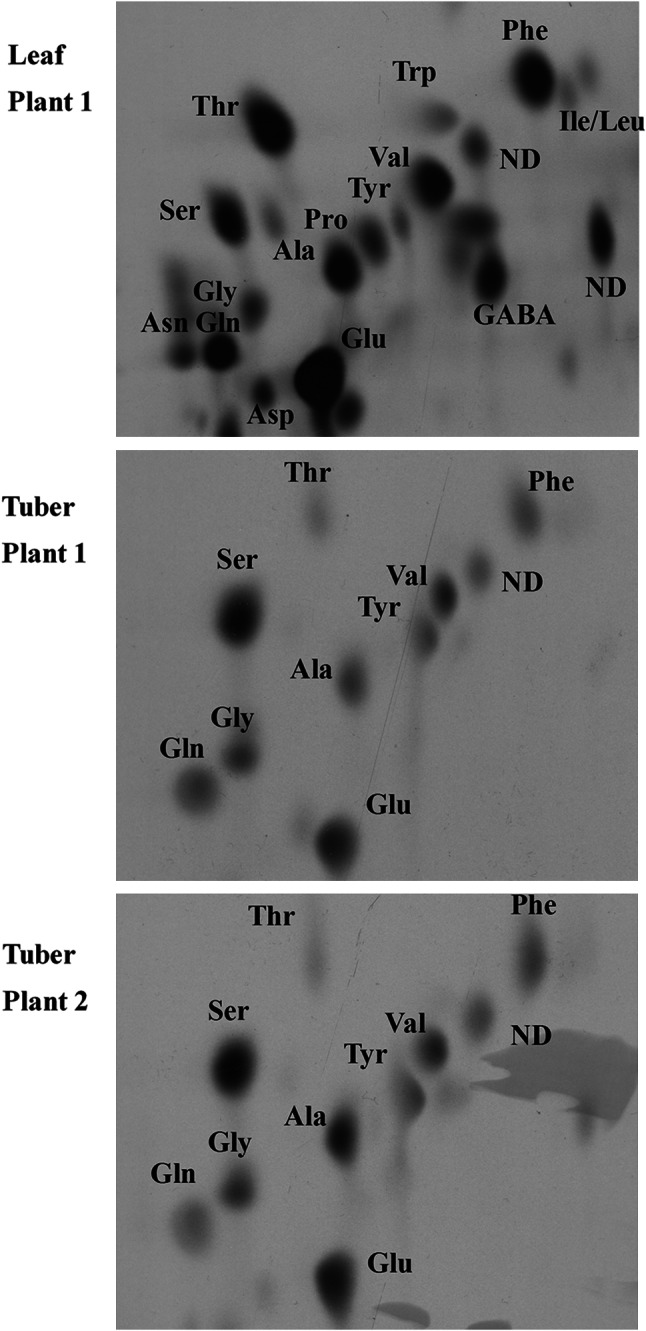
Autoradiographs of free amino acids from potato (*Solanum tuberosum*) cv. Saturna after assimilation of ^14^CO_2_. The free amino acids were purified and separated by thin-layer chromotography. Results are shown for an assimilating leaf from one plant and for tubers from the same plant and a second plant, as indicated

The separation of authentic standards of asparagine, glutamine and aspartate and, separately, of glutamine, aspartate and glutamate in the two-dimensional system chosen is shown in Fig. 1 by ninhydrin staining. The upper panel shows asparagine staining orange–brown, to the left of glutamine; the lower panel shows glutamine to the left and above glutamate and aspartate, with all three of these separated. These were the critical separations needed for the investigation. From Figs. 2 and 3, it is evident that this group of two di-carboxylic amino acids and their amides were separated from many other amino acids of interest.

### Separation of ^14^C-labelled amino acids from potato extracts

Figure 2 shows chromatograms stained with ninhydrin and the corresponding autoradiograms for extracts of the assimilating leaf, stem, stolon and tuber from the first experiment. The faint orange–brown staining of asparagine showed for all the tissues, but radioactivity in asparagine was clear on the autoradiograms only for extracts of the leaf and stem. Glutamine and glutamate were clearly the main amino acids labelled in the stolons and tubers, but many other amino acids were also more strongly labelled than asparagine. The amino acids staining with ninhydrin in the tuber extracts were glutamine, glutamate, aspartate, phenylalanine, valine, serine, threonine, alanine, GABA, lysine/histidine, glycine, proline and asparagine (Fig. 2). Asparagine is the most abundant amino acid in mature tubers (Eppendorfer and Bille 1996; Oruna-Concha et al. 2001; Amrein et al. 2003; Elmore et al. 2007; Halford et al. 2012a; Muttucumaru et al. 2013) but since ninhydrin staining is not a quantitative measure of amino acids the fact that asparagine was not heavily stained was not remarkable.

Figure 3 shows autoradiograms for leaf and tuber extracts from the experiment where ^14^CO_2_ was supplied separately to all the leaves of two single-stem potato plants. Results for a single leaf sample from one of the plants and single tuber samples from each plant are shown, but these were typical of a total of five different leaf samples and five tuber samples that were analysed. While asparagine was clearly labelled in the leaf extract, there was no radioactivity showing in asparagine in the tuber extracts. Glutamate, serine and alanine were strongly labelled in these tubers, with significant radioactivity also detectable in glutamine, glycine, tyrosine, valine, threonine and phenylalanine.

### Measurement of ^14^C incorporation into amino acids by scintillation counting

Scintillation counting of cellulose ‘spots’ scraped from a ninhydrin-stained chromatogram was undertaken and the results for glutamine, glutamate, asparagine, serine and alanine for both experiments are given in Table 2. In each case, the ^14^C content of all of the amino acids was lower in the stem, stolon and tuber samples than in the leaf, but the relative amounts of ^14^C present in each amino acid in the tuber broadly reflected the relative amounts in the leaf. In experiment 1, glutamine and glutamate both contained approximately tenfold the quantity of ^14^C that was present in asparagine. In the second experiment there was approximately five times the amount of radioactivity in glutamate than glutamine, with serine and alanine labelled to a similar extent to glutamine. The amount of ^14^C that was present in glutamate in this experiment was approximately 30 times that in asparagine. The differences between the two experiments in the relative labelling of amino acids may reflect the different conditions under which the plants were grown and the age of the plants when the assimilation and analysis took place.

**Table 2:** Incorporation of ^14^C into amino acids in the assimilating leaves, stems, stolons and tubers of potato (*Solanum tuberosum*) cv. Saturna plants after assimilation of ^14^CO_2_ by the leaf or leaves at the top of the plant

	^14^C (Bq)				
	Gln	Glu	Asn	Ser	Ala
Experiment 1					
Assimilating leaf	62.19	68.8	6.49	9.63	21.34
Stem below leaf	4.89	1.92	1.04	1.07	0.58
Stolon	1.17	0.63	0.16	0.22	0.30
Tuber	6.78	7.96	0.75	3.79	2.45
Experiment 2					
Plant 1					
Assimilating leaves	1,114	5,428	176	1,068	1,086
Stem	49	187	4	148	21
Stolon	11	15	4	13	2
Tuber 1	21	31	6	48	10
Experiment 2					
Plant 2					
Assimilating leaves	2,638	14,106	470	2,446	1,688
Stem	31	98	3	89	18
Stolon	5	21	1	13	2
Tuber 1	96	160	20	135	74
Tuber 2	34	124	2	80	25

The amino acids were separated by thin-layer chromatography and stained with ninhydrin. The cellulose containing each amino acid was then scraped off and ‘counted’ in a scintillation analyser. Results are given for glutamine, glutamate, serine and alanine, which were the major transported amino acids, and for asparagine

The statistical method of REML was used to fit a linear mixed model to the data from the second experiment, since this experiment provided replication but also had two tuber samples from one plant (Table 2); this imbalance precluding a usual analysis of variance (ANOVA). A natural log (to base *e*) transformation was applied to the data to account for heterogeneity of variance across the tissue by amino acid combinations and the means are given in (Table 3), along with the average standard error of the difference (SED) and least significant difference (LSD) (5 %) for making comparisons. The analysis showed there to be significantly (*P* < 0.05, LSD) less ^14^C in asparagine than the other amino acids in all tissues except the stolon, where the figures for asparagine and alanine were not significantly different but were significantly (*P* < 0.05, LSD) lower than for the other amino acids.

**Table 3:** Means on the natural log (to base *e*) scale from the data shown in Table 2 for Experiment 2

	^14^C (Bq)				
	Gln	Glu	Asn	Ser	Ala
Assimilating leaves	7.447	9.077	5.662	7.388	7.211
Stem	3.663	4.908	1.242	4.743	2.967
Stolon	2.004	2.876	0.693	2.565	0.693
Tuber	3.712	4.443	1.827	4.386	3.275
Average SED = 0.5951 on 20 *df*; Average LSD (5 %) = 1.2414					

There was significantly (*p* < 0.05, LSD) less Asn than other amino acids for all tissues except the stolon, where Asn and Ala were least

## Discussion

The aim of this study was to assess the importance of import as opposed to in situ synthesis in the accumulation of free asparagine in potato tubers and to identify the major amino acids transported from leaf to tuber. Asparagine is the major free amino acid of potato tubers and its accumulation represents a problem for the food industry because it forms the contaminant, acrylamide, when it reacts with carbonyl compounds in the final stages of the Maillard reaction.

Main crop potatoes are normally harvested when the haulms are dying back so that by then the main supply of sugar and amino acids is complete and changes in tuber composition depend on a ripening process. This study, therefore, focused on translocation while the leaves were green and the tubers were young and developing. The method used was to monitor the incorporation of ^14^C into free amino acids after its assimilation by a leaf or, in a second experiment, leaves of potato plants. Free amino acids were purified, separated by two-dimensional TLC and identified by their migration position and colour after staining with ninhydrin. The presence of ^14^C in individual amino acids was demonstrated by autoradiography of a TLC plate and the relative amounts of ^14^C in asparagine compared to the major transported amino acids (glutamine, glutamate, serine and alanine) were obtained by scintillation counting.

Photosynthetic assimilation of ^14^CO_2_ has been used from the time the radiochemical first became available in the 1940s to trace the metabolic intermediates involved in carbon assimilation in leaves and algae (Benson 2002; Bassham 2003) and the pathways of subsequent synthesis of many plant constituents, including starch, sucrose, hexoses and protein (Porter and Martin 1952; Ongun and Stocking 1965). It has been used to trace the path of export of photosynthate from leaves to tubers in potato (Moorby 1968; Oparka and Davies 1985) but to our knowledge has not been used previously to identify the amino acids that are transported. Here, incorporation of ^14^C into amino acids in the assimilating leaf was readily detectable and ^14^C-labelled amino acids were also identified in the stem, stolon, and the tuber developing directly beneath a single assimilating leaf, and more widely when multiple leaves were involved and more ^14^C was assimilated.

Amino acids that were labelled in the stem, stolon and tuber were also labelled in the assimilating leaf. A relatively short time was deliberately allowed for transport to avoid confusion with carbon incorporated into amino acids via the organic acids derived from the respiration of sugars in the tuber. The result is consistent with the general view of phloem loading and unloading of amino acids, which accepts that the amino acid composition of the phloem sap reflects the concentrations in the leaves and the composition available to storage organs and growing tissues. Lohaus and Fischer (2002), for example, discussed the similarity of the relative concentrations of amino acids in the phloem with the concentration in the mesophyll cells in barley leaves. However, this consensus view does not exclude the possibility of some variation between species due to the route by which the transported substances are moved from the mesophyll cells to the vessels (Oparka and Turgeon 1999).

When assimilation of ^14^C was by a single leaf, scintillation counting of crude extracts found no evidence of transport of labelled compounds from the assimilating leaf to other leaves or roots, or to tubers other than the tuber immediately below the leaf. Crude extracts would contain sucrose, suggesting that the transport of sucrose and amino acids from the fed leaf followed similar pathways and did not reach all parts of the plant. Oparka and Davies (1985) also concluded that translocation in potato was compartmentalised, with little exchange of assimilated carbon between stems.

Most importantly, this study found no evidence to suggest that asparagine is a major transport amino acid from leaf to tuber in potato. Instead, the predominant amino acids imported into the tuber from the ^14^C-assimilating leaf in the first experiment were glutamine and glutamate, with phenylalanine, valine, serine, threonine, GABA and alanine also detectable. In the second experiment, in which more ^14^C was used and the plants were grown under different conditions, proline, tyrosine and glycine were also detectable, with serine and glutamate predominant. Only one variety was analysed and extending the study to more varieties was considered unfeasible given the amount of ^14^C-contaminated waste that would be produced. However, Saturna is a current commercial variety in the United Kingdom and there is no reason to expect it to be atypical. Furthermore, the data are consistent with the results of a study by Karley et al. (2002), which showed that glutamine and glutamate, not asparagine, were the most abundant amino acids in potato phloem sap, although that study did not analyse amino acids accumulating in the tuber. The conclusions are also in agreement with inferences drawn from studies in which asparagine synthetase gene expression in the tubers of transgenic potato plants has been reduced by RNA interference (Rommens et al. 2008; Chawla et al. 2012).

In conclusion, the study demonstrated that glutamine, glutamate and serine are the major transport amino acids from leaf to tuber in potato, with alanine, aspartate, GABA, glycine, phenylalanine, proline, threonine and valine also playing a part. It found no evidence for asparagine being a major transport amino acid in potato, and the high concentrations of free asparagine that accumulate in potato tubers must, therefore, arise from synthesis in situ rather than import. Genetic interventions to reduce free asparagine concentration in potato tubers will, therefore, have to target asparagine metabolism specifically in the tuber. The fact that asparagine does not play a major role in the transport of nitrogen in potato makes potato a somewhat unusual plant species.

## Abbreviations

GABA: γ-Aminobutyric acid
TLC: Thin-layer chromatography
